# The Relationship Between Internal Stigmatisation, Recovery and Treatment Adherence in Individuals with Schizophrenia

**DOI:** 10.1192/j.eurpsy.2024.1522

**Published:** 2024-08-27

**Authors:** M. S. Keskiner, S. Aktaş, M. C. Aktaş, H. Ayhan, K. Aslan

**Affiliations:** ^1^Department of Pyschiatric Nursing, Yüzüncü Yıl University, Faculty of Health Sciences; ^2^Department of Pyschiatric Nursing, Health Sciences University Van Training and Research Hospital, Van, Türkiye

## Abstract

**Introduction:**

Schizophrenia has a clinical course that has a great negative impact on the daily life of the person due to the cognitive and social problems it causes. Internalised stigmatisation is a very common negative phenomenon in individuals diagnosed with schizophrenia. It is known that treatment adherence is low in schizophrenia patients with high levels of internalised stigma. Lack of adequate treatment adherence in these patients is a negative factor in terms of recovery. Reducing the level of internal stigmatisation and reinforcing treatment adherence in schizophrenia has a positive effect on recovery. Considering this situation, it is important to determine the relationship between internal stigmatisation, treatment adherence and recovery in schizophrenia patients. In the literature review, there were no studies in which the relationship between internal stigmatisation, recovery and treatment adherence in individuals diagnosed with schizophrenia was carried out together.

**Objectives:**

In this study, it was aimed to fill the existing gap in the relevant field and to be a resource for further intervention programmes.

**Methods:**

The study was planned as descriptive. The sample of the study consisted of individuals diagnosed with schizophrenia aged 18 years and over who met the inclusion criteria and accepted to participate in the study by purposive sampling method. In the power analysis, the sample number was calculated as (N=80) with a margin of error of 0.5. Personal information form, Internalised Stigma Scale in Mental Illness (ISMI), Recovery Assessment Scale (RAS) and Medication Adherence Rating Scale (MARS) were used for data collection. IBM SPSS 27.0 package programme was used for statistical analysis.

**Results:**

The data are still being analysed in detail by the researchers. The findings and relational results of the study will be presented.

**Conclusions:**

It is thought that the results of the study will contribute to the reporting of the relationship between intrinsic stigma, recovery and treatment adherence in individuals diagnosed with schizophrenia, and by revealing the relationship between the variables, it is thought that it will be a source for planning interventions that will increase the treatment adherence and recovery perceptions of schizophrenia patients and reduce their intrinsic stigma.

**Disclosure of Interest:**

None Declared

